# Mitochondrial DNA Provides a Link between Polynesians and Indigenous Taiwanese

**DOI:** 10.1371/journal.pbio.0030281

**Published:** 2005-07-05

According to folklore, Polynesians originated from a mythical homeland called Hawaiki. The existence of such a place, however, as well as its location, has been the subject of much speculation. Significant research efforts have attempted to elucidate these claims through archeological, linguistic, and, more recently, biological evidence. Jean Trejaut and colleagues are among the research groups attempting to determine the link between Polynesians and other Southeast Asian populations. In their recent study, they provide evidence that indigenous Taiwanese and Polynesians share a common ancestral link.

Two main theories have previously emerged to explain the origins of modern day Polynesians: the express train model and the slow boat model. The express train model proposes that early ancestors migrated from mainland China and Southeast Asia, colonizing Taiwan first and then spreading rapidly to the other Pacific Islands. The slow boat model assumes that Polynesian culture was influenced by gradual, complex interactions with neighboring islands before reaching Polynesia.

Recently, genetic techniques involving mitochondrial DNA (mtDNA) have been used to compare the genetic profiles of Polynesians with people from mainland China, Southeast Asia, and Taiwan. Mitochondria reside in the cell cytoplasm and contain separate DNA that is inherited only from the mother. This means that while a person's nuclear DNA comes from a large number of ancestors, mtDNA can be traced back to a single ancestor. In theory, every person should have a copy of mtDNA identical to this original ancestor. In practice, this is not the case because random errors occur in the replication process. Different populations will experience mutations at different locations in their mtDNA, and these will be passed on to future generations. The result is that some groups of people will end up with mtDNA that is very different from another group. By comparing how much mtDNA different populations have in common, an ancestral relationship can be determined and dated. [Fig pbio-0030281-g001]

**Figure pbio-0030281-g001:**
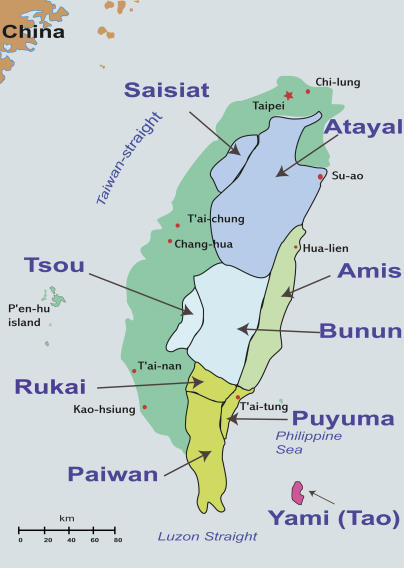
Ancient mitochondrial DNA from these nine indigenous Taiwanese tribes provided clues about their movement throughout Polynesia

Early results from studies using mtDNA to explore this question were conflicting or inconclusive; however, recent research by Trejaut et al. has shed more light on the subject. Trejaut et al. analyzed DNA from people in China, Southeast Asia, Polynesia, and Taiwan. The authors focused specifically on the aboriginal populations of Taiwan, thought to be ancestors of today's Polynesians, and looked for unique genetic markers that occurred in the aboriginal people. They then compared these markers to those found in mainland Chinese, Taiwanese, and other Southeast Asian peoples, and asked: do the aboriginal people of Taiwan have a common ancestor in mainland China, and if yes, how long ago? And do the aboriginal Taiwanese share a common ancestry with the Polynesians?

Although Taiwan is currently inhabited mainly by migrants of recent Chinese origin, this has not always been the case. Today, roughly 2% of the inhabitants are direct descendents of the island's indigenous people and have a unique culture, language, and genetic makeup. And while the Chinese immigrants colonized Taiwan a mere 400 years ago, archeological records show that Taiwan may have been inhabited for the last 15,000 years.

Trejaut et al. found that the indigenous Taiwanese, Melanesian, and Polynesian populations share three specific mutations in their mtDNA that do not occur in mainland east Asian populations. Furthermore, they showed that there were enough different mtDNA mutations between the mainland Chinese population and the aboriginal Taiwanese to support the archeological findings suggesting a long period of habitation.

Taken together, these results suggest that Taiwanese aboriginal populations have genetically been isolated from mainland Chinese for 10,000 to 20,000 years, though the whereabouts of their origin in the Asian region is still unclear. Additionally, these results demonstrate that Polynesian migration most likely originated from people identical to the aboriginal Taiwanese. These findings provide the first direct evidence for the common ancestry of Polynesians and indigenous Taiwanese, and suggest that Taiwan genetically belongs to that region of insular Southeast Asia that might have been the point from where Polynesians started their migration across the Pacific, followed by later cultures that developed from their descendents in east Indonesia and Melanesia. Further research will be necessary to accurately determine the origins of the aboriginal Taiwanese; however, these results are a step towards clarifying the origins of Polynesians.

